# Entropy-Based Dual-Teacher Distillation for Efficient Motor Imagery EEG Classification

**DOI:** 10.3390/e28030310

**Published:** 2026-03-10

**Authors:** Zefeng Xu, Zhuliang Yu

**Affiliations:** School of Automation Science and Engineering, South China University of Technology, Guangzhou 510641, China; auzefengxu@mail.scut.edu.cn

**Keywords:** motor imagery (MI), brain–computer interface (BCI), knowledge distillation, ensemble learning, EMA teacher, predictive entropy

## Abstract

Motor imagery (MI) EEG classification is a key component of noninvasive brain–computer interfaces (BCIs) and often must satisfy strict latency constraints in online or edge deployments. Although ensembling can reliably improve MI decoding accuracy, its inference cost grows linearly with the number of ensemble members, making it impractical for low-latency applications. To address these issues, we propose an entropy-based dual-teacher distillation framework that transfers ensemble teacher knowledge to a single deployable backbone. From an information theoretic perspective, two failure modes are common in small and noisy MI datasets: elevated predictive entropy (noisy decisions) and large fluctuation across late training epochs (unstable convergence and unreliable checkpoint selection). Thus, we introduce an exponential moving average (EMA) teacher with entropy-gated activation as a low-pass filter in parameter space to reduce the student’s prediction noise. In addition, a two-stage cosine annealing schedule is employed to suppress late-stage oscillations and improve the robustness of final checkpoint selection. Experiments on two public MI benchmarks (BCI Competition IV-2a and IV-2b) with three representative backbones (EEGNet, ShallowConvNet, and ATCNet) under the subject dependent protocol show consistent accuracy gains over the ensemble teacher and strong distillation baselines. On IV-2a, our method achieves an average accuracy of 0.7713 across the backbones, surpassing both the original models (0.7222) and the corresponding ensembles (0.7482); on IV-2b, it achieves 0.8583 versus 0.8432 (original) and 0.8529 (ensemble).

## 1. Introduction

Motor imagery (MI) EEG classification is a core component of noninvasive brain–computer interfaces (BCIs) and is often required to operate under strict latency and computing constraints in online or edge deployments [1,2]. Despite substantial progress, EEG-based decoding remains challenging due to the low signal-to-noise ratio (SNR) [3,4]. In MI, the problem is further aggravated by limited labeled data per subject and pronounced inter-subject and inter-session variability [5,6]. As a result, training a single model can be sensitive to random initialization and optimization noise, leading to unstable generalization and unreliable final checkpoint selection in practice [7].

Ensembling is a reliable way to improve accuracy and reduce variance, but its inference cost scales linearly with the number of ensemble members, which is undesirable for real-time BCI systems. Knowledge distillation (KD) addresses this “train heavy, infer light” objective by transferring an ensemble’s predictive distribution to a single student model [8]. However, in MI-EEG settings, student optimization can be noisy, and a fixed teacher alone may not prevent instability during optimization.

From an information theoretic perspective, two failure modes are particularly common in small and noisy MI datasets. First, the predicted class distribution can remain noisy, resulting in elevated predictive entropy and thus uncertain decisions. Second, even when the accuracy improves, the predictive distribution may oscillate noticeably across epochs near convergence, leading to large entropy fluctuation and unstable final checkpoint selection. These two phenomena motivate a stability-oriented training strategy, where predictive entropy serves as a measurable proxy for the reliability of the predictive distribution: we aim to make predictions less noisy and more consistent, while suppressing late-stage oscillations near convergence.

To address the above issues, we propose an entropy-based dual-teacher distillation framework that combines (i) an offline ensemble teacher built from multiple instances of a backbone network and (ii) an entropy-gated exponential moving average (EMA) teacher formed from the student’s historical weights [9]. The ensemble teacher provides high-quality soft targets for transferring strong decision boundaries. Meanwhile, the EMA teacher plays a complementary role as a low-pass filter over the student’s historical weights, producing temporally smoothed teacher logits that reduce target noise, which is activated based on the sample’s current predictive entropy to allocate stronger EMA guidance to high-entropy samples and down-weights low-entropy samples, concentrating the stabilization effect where it is most needed. We further adopt a two-stage cosine annealing schedule [10] to suppress entropy fluctuation in the late training stage, improving the robustness of final checkpoint selection when early stopping is not used.

Our main contributions are as follows:

1. We propose an entropy-based dual-teacher distillation framework for MI EEG that distills an offline ensemble into a single deployable backbone student without increasing the inference time.
2. We introduce an EMA teacher as a parameter-space low-pass filtering mechanism that yields more stable teacher logits. It is activated based on the sample’s predictive entropy to concentrate this effect on high-noise samples and avoid redundant regularization on easy samples.
3. We integrate a two-stage cosine annealing schedule to suppress late-stage entropy fluctuation, yielding more stable training dynamics and more reliable final checkpoint selection.
4. We evaluate the proposed method on BCI Competition IV-2a/2b with three representative backbones, and provide entropy-based analyses to link the accuracy gains to the improved reliability of predictive distributions [11,12].

## 2. Related Work

Classical MI decoding commonly relies on spatial filtering and linear classification, such as CSP/FBCSP pipelines [6,13,14]. Geometry-aware approaches based on covariance representations on Riemannian manifolds have also been studied [15,16]. Deep learning has become the dominant approach for end-to-end MI decoding. Widely used CNN architectures include ShallowConvNet [17] and EEGNet [18]. Recent models incorporate stronger temporal modeling [19,20], attention mechanisms [21,22,23], and state–space sequence models [24]. A recent survey further systematizes deep learning-based MI-EEG research by summarizing input formulations, architectures, and commonly used public datasets [25]. While these architectures improve accuracy, MI training data remain scarce and noisy, making training stability central concerns for further improvement [7].

KD transfers soft targets from a teacher (often a larger network) to a student and is a standard tool for compressing knowledge into a single deployable model [8]. Several variants are closely related to our setting: Deep Mutual Learning (DML) uses peer networks for online knowledge exchange [26]; Born-Again Networks (BAN) iteratively distill a model into a new instance of the same architecture [27]; and Decoupled KD (DKD) refines logit distillation by separating target-class and non-target-class components [28].

Distillation has been applied to MI decoding. Examples include multi-subject or cross-subject distillation strategies [29,30], relation/similarity-preserving distillation for low-density EEG [31], and more structured teacher–assistant designs targeting high compression [32]. Self distillation is also studied [33]. These studies support the application of distillation in MI-BCI, but many require additional intermediate networks or focus on scenario-specific constraints. In contrast, our approach uses an ensemble of the same backbone as teacher, while keeping the inference time identical to the backbone student. No additional teacher or assistant network architecture is required, which is particularly useful when the backbone is already strong, and a strictly stronger single-model teacher or assistant is hard to obtain.

Predictive uncertainty and training stability can be naturally described from an information theoretic perspective. Given a probabilistic classifier, the Shannon entropy of its output distribution provides a measure of decision uncertainty, where higher entropy indicates a flatter prediction [11]. Calibration and reliability are practically relevant to performance. This motivates calibration diagnostics such as reliability diagrams and the expected calibration error (ECE) [12,34], as well as complementary proper scoring rules (e.g., the Brier score) for assessing probabilistic forecasts [35]. Meanwhile, uncertainty quality and robustness are known to benefit from model averaging: deep ensembles often reduce overconfidence [36]. Bayesian approximations such as Monte Carlo Dropout are widely used to quantify uncertainty in deep networks [37,38]. Beyond calibration, predictive entropy is also informative about the stability of the predictive distribution during optimization [39,40]: non-common-mode perturbations that change relative logit differences tend to yield less consistent predictions and are often reflected by higher entropy on average.

## 3. Methods

### 3.1. Problem Formulation and Notation

We consider supervised MI EEG classification with *C* classes, where *C* denotes the number of MI categories (e.g., left/right hand, feet, tongue, etc.). Each trial is denoted by (x,y), where $x\in R^{N_{ch}\times T}$ is the multi-channel EEG segment, $N_{ch}$ is the number of channels, *T* is the number of time points, and y∈{1,…,C} is the class label. Let $D_{tr}={\left\{\left(x_{i},y_{i}\right)\right\}}_{i=1}^{n_{tr}}$ and $D_{te}={\left\{\left(x_{j},y_{j}\right)\right\}}_{j=1}^{n_{te}}$ denote the training and test sets under a chosen evaluation protocol, where $n_{tr}$ and $n_{te}$ are the numbers of training and test trials, respectively.

We use a single backbone network as the student S(·;θ), where θ represents all trainable parameters, producing logits $z_{S}=S\left(x;\theta \right)\in R^{C}$ and class probabilities

(1) $$p_{S}=\sigma \left(z_{S}\right)=\mathrm{softmax}\left(z_{S}\right).$$

During training, we employ two teachers: (i) an ensemble teacher $T_{\mathrm{ens}}$ (trained offline, fixed during student training), producing $z_{\mathrm{ens}}$$\in R^{C}$ and $p_{\mathrm{ens}}$$=\mathrm{softmax}(z_{\mathrm{ens}})$; (ii) an EMA teacher $T_{\mathrm{ema}}$ (trained online, updated alongside student training), producing $z_{\mathrm{ema}}$$\in R^{C}$ and $p_{\mathrm{ema}}$$=\mathrm{softmax}(z_{\mathrm{ema}})$. Importantly, only the student is used at the inference time, both teachers are only used during the training time.

### 3.2. Teacher Construction

#### 3.2.1. Ensemble Teacher via K-Fold Bagging

To obtain a strong teacher when a strictly stronger single-model architecture is unavailable, we construct an ensemble teacher using *K* independently trained instances of the same backbone. We split the training set $D_{tr}$ into *K* disjoint folds:

(2) $$D_{tr}=\overset{K}{\underset{k=1}{⋃}}D_{k},\;D_{i}\cap D_{j}=\emptyset \;\left(i\neq j\right).$$

For teacher *k*, we train on $D_{tr}\setminus D_{k}$ to encourage diversity. After training, each teacher yields logits $z^{\left(k\right)}\left(x\right)$. The ensemble logits are computed by averaging

(3) $$z_{\mathrm{ens}}\left(x\right)=\frac{1}{K}\sum_{k=1}^{K}z^{\left(k\right)}\left(x\right).$$

We then obtain the ensemble soft target at temperature τ>0:

(4) $$q_{\mathrm{ens}}\left(x\right)=\sigma \;\left(\frac{z_{\mathrm{ens}}\left(x\right)}{\tau }\right).$$

#### 3.2.2. EMA Teacher

We maintain an EMA of student parameters as an additional teacher. Let $\theta_{\mathrm{ema}}$ be the EMA parameters and α∈[0,1) the smoothing coefficient. After each student update, we perform

(5) $$\theta_{\mathrm{ema}}\leftarrow \alpha \;\theta_{\mathrm{ema}}+\left(1-\alpha \right)\;\theta .$$

This update can be viewed as a low-pass filter in parameter space, which attenuates high-frequency optimization noise and yields temporally smoothed teacher logits. The EMA teacher produces logits $z_{\mathrm{ema}}=S\left(x;\theta_{\mathrm{ema}}\right)$ and a temperature-scaled soft target

(6) $$q_{\mathrm{ema}}\left(x\right)=\sigma \;\left(\frac{z_{\mathrm{ema}}\left(x\right)}{\tau }\right).$$

### 3.3. Entropy-Based Dual-Teacher Distillation Objective

We use the standard cross-entropy as the supervised classification loss:

(7) $$L_{\mathrm{ce}}\left(x,y\right)=-\log p_{S}\left[y\right].$$

$p_{S}\left[y\right]$ is the *y*th element of vector $p_{S}$. The ensemble knowledge is distilled by matching temperature-scaled distributions via KL divergence:

(8) $$L_{\mathrm{kd}}^{\mathrm{ens}}\left(x\right)=\tau^{2}\;\mathrm{KL}\;\left(q_{\mathrm{ens}}\left(x\right)\;∥\;\sigma \;\left(\frac{z_{S}\left(x\right)}{\tau }\right)\right).$$

Similarly, we align the student to the EMA teacher:

(9) $$L_{\mathrm{kd}}^{\mathrm{ema}}\left(x\right)=\tau^{2}\;\mathrm{KL}\;\left(q_{\mathrm{ema}}\left(x\right)\;∥\;\sigma \;\left(\frac{z_{S}\left(x\right)}{\tau }\right)\right).$$

We introduce a sample level entropy gate w(x) that modulates the EMA KD term. Specifically, we compute the predictive entropy of the EMA teacher on x:

(10) $$H_{\mathrm{ema}}\left(x\right)=-\sum_{c=1}^{C}q_{\mathrm{ema}}\left(x\right)\left[c\right]\log q_{\mathrm{ema}}\left(x\right)\left[c\right],$$

and then normalize the entropy into [0,1] as

(11) $$h\left(x\right)=\frac{H_{\mathrm{ema}}\left(x\right)}{\log C}.$$

The entropy gate is defined as a linear and clipped mapping:

(12) $$w\left(x\right)=\mathrm{clip}\;\left(\frac{h\left(x\right)-h_{\mathrm{low}}}{h_{\mathrm{high}}-h_{\mathrm{low}}},\;0,\;1\right),$$

where $0\leq h_{\mathrm{low}}<h_{\mathrm{high}}\leq 1$ are two hyperparameters. This design assigns a smaller EMA weight to high-entropy (eazy) samples and a larger EMA weight to high-entropy (noisy) samples.

The overall objective is

(13) $$L=L_{\mathrm{ce}}+\lambda_{\mathrm{ens}}\;L_{\mathrm{kd}}^{\mathrm{ens}}+\lambda_{\mathrm{ema}}\left(e\right)\;w\left(x\right)\;L_{\mathrm{kd}}^{\mathrm{ema}},$$

where *e* is the epoch index, and $\lambda_{\mathrm{ens}}$ and $\lambda_{\mathrm{ema}}\left(e\right)$ control the contributions of the two teachers.

### 3.4. Two-Stage Cosine Annealing Schedule

MI-EEG training often exhibits noticeable late-stage oscillations. We adopt cosine annealing to reduce oscillation. Let $\eta_{\max}$ and $\eta_{\min}$ be the maximum and minimum learning rates. Within a stage of length $T_{s}$, the learning rate at step $t\in [0,T_{s}]$ is

(14) $$\eta \left(t\right)=\eta_{\min}+\frac{1}{2}\left(\eta_{\max}-\eta_{\min}\right)\left(1+\cos\left(\pi \frac{t}{T_{s}}\right)\right).$$

We use a two-stage schedule:

- Phase I (length *N* epochs): Train the student with $L_{\mathrm{ce}}+\lambda_{\mathrm{ens}}L_{\mathrm{kd}}^{\mathrm{ens}}$, i.e., $\lambda_{\mathrm{ema}}\left(e\right)=0$.
- Phase II (after restart, length 2N epochs): Enable EMA guidance and train with $L_{\mathrm{ce}}+\lambda_{\mathrm{ens}}L_{\mathrm{kd}}^{\mathrm{ens}}+\lambda_{\mathrm{ema}}w\left(x\right)L_{\mathrm{kd}}^{\mathrm{ema}}$, i.e., $\lambda_{\mathrm{ema}}\left(e\right)>0$.

**Figure 1:**
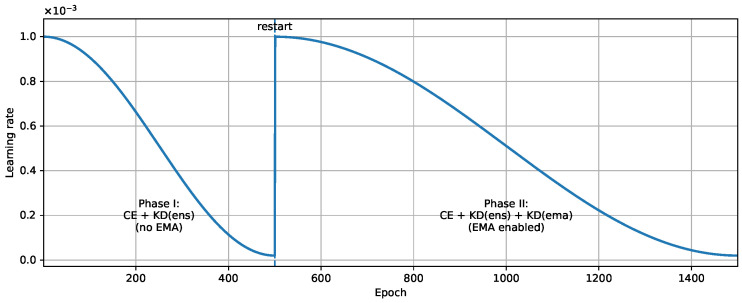
Two-stage training schedule with cosine annealing and EMA activation. *N* is set to 500.

Figure 1 shows the two-stage training schedule with cosine annealing and EMA activation. This design is motivated by the fact that the EMA teacher is low quality at the beginning (since it is derived from an untrained student). Delaying EMA activation prevents early-stage noisy targets.

### 3.5. Training Procedure

Algorithms 1 and 2 summarize the complete training pipeline. Algorithm 1 constructs the ensemble teacher by training *K* backbone teachers using a K-fold scheme on the training set $D_{tr}$. Specifically, $D_{tr}$ is partitioned into *K* disjoint folds ${\left\{D_{k}\right\}}_{k=1}^{K}$. Each teacher $T^{\left(k\right)}$ is trained on $D_{tr}\setminus D_{k}$ for the same number of epochs $E_{T}$ using the cross-entropy (CE) loss only. After all teachers are trained, the ensemble teacher prediction $z_{ens}\left(x\right)$ for an input x is obtained by averaging teacher logits, which is later converted into a temperature-scaled soft target $q_{ens}\left(x\right)$ during student training.
**Algorithm 1** Offline Training of K-fold Teachers**Require:** Training set $D_{tr}$; backbone network S(·;θ); number of folds/teachers *K*; teacher epochs $E_{T}$ (same for all teachers); teacher learning rate $\eta_{T}$. **Ensure:** Trained teachers ${\left\{T^{\left(k\right)}\right\}}_{k=1}^{K}$. 1:Split $D_{tr}$ into *K* disjoint folds ${\left\{D_{k}\right\}}_{k=1}^{K}$.2:**for** k←1 **to** *K* **do**3:      Initialize teacher parameters $\theta^{\left(k\right)}$.4:      **for** e←1 **to** $E_{T}$ **do**5:            **for all** batches $B\subset (D_{tr}\setminus D_{k})$ **do**6:                   Compute the batch CE loss $L_{\mathrm{CE}}^{\left(k\right)}$7:                   Back propagate $L_{\mathrm{CE}}^{\left(k\right)}$8:                   Update $\theta^{\left(k\right)}$ with learning rate $\eta_{T}$9:            **end for**10:      **end for**11:      Store trained teacher $T^{\left(k\right)}\left(\cdot \right)≜S\left(\cdot ;\theta^{\left(k\right)}\right)$.12:**end for**13:**return** ${\left\{T^{\left(k\right)}\right\}}_{k=1}^{K}$.


Algorithm 2 trains the deployable student under a two-stage cosine annealing schedule with a single restart. For epoch e∈{1,…,3N}, the learning rate η(e) is computed by mapping *e* to the local index *t* and stage length $T_{s}$: $t=e-1,\;T_{s}=N$ for Phase I (e≤N), and $t=e-1-N,\;T_{s}=2N$ for Phase II (e>N). η(e) is then computed by cosine annealing. For each minibatch, the student first computes logits $z_{S}\left(x\right)$ and supervised CE loss $L_{ce}$. Next, the ensemble KD term is computed by forming $z_{ens}$ and $q_{ens}$ and then applying KL-based distillation. The EMA KD term is activated only in Phase II. At the beginning of Phase II (e=N+1), the EMA parameters are initialized as $\theta_{ema}\leftarrow \theta$, and for each subsequent minibatch, the EMA teacher produces logits $z_{ema}\left(x\right)$ and soft target $q_{ema}\left(x\right)$, which are used to compute the corresponding distillation loss $L_{kd}^{ema}$ and entropy gate w(x). Finally, the student is updated by minimizing the total objective L, and the EMA parameters are updated via $\theta_{ema}\leftarrow \alpha \theta_{ema}+\left(1-\alpha \right)\theta$.

**Figure 2:**
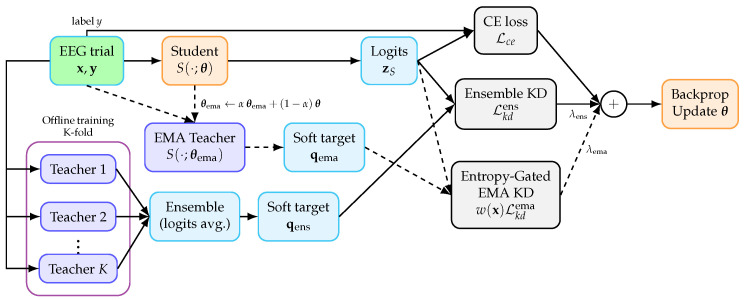
Overall framework of the proposed dual-teacher distillation. The ensemble teacher is trained offline using *K* backbone teachers (CE-only, K-fold/bagging) and provides $q_{\mathrm{ens}}$ for ensemble KD. The EMA teacher is activated only in Phase II (indicated by dashed connections) and provides $q_{\mathrm{ema}}$ for the entropy-gated EMA KD. The student is optimized by summing three losses, and backpropagation updates only the student parameters. Only the student is used for inference.

Figure 2 provides an overview of the proposed training framework that corresponds to Algorithm 2.
**Algorithm 2** Online Student Training with Dual-Teacher Distillation**Require:** Training set $D_{tr}$; backbone network S(·;θ); trained teachers ${\left\{T^{\left(k\right)}\right\}}_{k=1}^{K}$; number of teachers *K*; student phase length *N* (total epochs =3N); temperature τ; EMA coefficient α; learning-rate bounds $\left(\eta_{\max},\eta_{\min}\right)$; KD weights $\lambda_{\mathrm{ens}}$ and $\lambda_{\mathrm{ema}}$. **Ensure:** Deployable student parameters θ.1: **Definitions:**2: σ(z)≜softmax(z)3: $\sigma_{\tau }\left(z\right)≜\mathrm{softmax}\left(z/\tau \right)$4: $\mathrm{KL}\left(a∥b\right)≜\sum_{c=1}^{C}a_{c}\log\frac{a_{c}}{b_{c}}$5: $\mathrm{KD}\left(q,z_{S}\right)≜\tau^{2}\cdot \mathrm{KL}\;\left(q\;∥\;\sigma_{\tau }\left(z_{S}\right)\right)$6: $T_{\mathrm{logit}}^{\left(k\right)}\left(x\right)$≜ Logits output of $T^{\left(k\right)}$ with input *x*7: w(x)≜ entropy gate of sample *x*8: Initialize student parameters θ.9: **for** e←1 **to** 3N **do**10:     **if** e≤N **then**11:           t←e−1,    $T_{s}\leftarrow N$▹ Phase I segment12:     **else**13:           t←e−1−N,    $T_{s}\leftarrow 2N$▹ Phase II segment (after restart)14:     **end if**15:     $\eta \left(e\right)\leftarrow \eta_{\min}+\frac{1}{2}\left(\eta_{\max}-\eta_{\min}\right)\left(1+\cos\left(\pi \frac{t}{T_{s}}\right)\right)$16:     **if** e=N+1 **then**17:           $\theta_{\mathrm{ema}}\leftarrow \theta$18:     **end if**19:     **for all** batches $B\subset D_{tr}$ **do**20:           **for all** (x,y)∈B **do**21:                 $z_{S}\left(x\right)\leftarrow S\left(x;\theta \right)$22:                 $p_{S}\left(x\right)\leftarrow \sigma \left(z_{S}\left(x\right)\right)$23:                 $L_{ce}\leftarrow -\log p_{S}\left(x\right)\left[y\right]$24:                 $z_{\mathrm{ens}}\left(x\right)\leftarrow \frac{1}{K}\sum_{k=1}^{K}T_{\mathrm{logit}}^{\left(k\right)}\left(x\right)$▹ teacher logits average25:                 $q_{\mathrm{ens}}\left(x\right)\leftarrow \sigma_{\tau }\;\left(z_{\mathrm{ens}}\left(x\right)\right)$▹ soft target26:                 $L_{kd}^{\mathrm{ens}}\leftarrow \mathrm{KD}\;\left(q_{\mathrm{ens}}\left(x\right),\;z_{S}\left(x\right)\right)$27:                 **if** e>N **then**28:                       $z_{\mathrm{ema}}\left(x\right)\leftarrow S\left(x;\theta_{\mathrm{ema}}\right)$29:                       $q_{\mathrm{ema}}\left(x\right)\leftarrow \sigma_{\tau }\;\left(z_{\mathrm{ema}}\left(x\right)\right)$30:                       $L_{kd}^{\mathrm{ema}}\leftarrow \mathrm{KD}\;\left(q_{\mathrm{ema}}\left(x\right),\;z_{S}\left(x\right)\right)$31:                       Compute w(x) with $q_{\mathrm{ema}}\left(x\right)$32:                 **else**33:                       $L_{kd}^{\mathrm{ema}}\leftarrow 0$34:                 **end if**35:                 $L\leftarrow L_{ce}+\lambda_{\mathrm{ens}}L_{kd}^{\mathrm{ens}}+\lambda_{\mathrm{ema}}w\left(x\right)L_{kd}^{\mathrm{ema}}$36:           **end for**37:           $L\leftarrow \frac{1}{\left|B\right|}L$38:           Back propagate L39:           Update θ with learning rate η(e)40:           **if** e>N **then**41:                 $\theta_{\mathrm{ema}}\leftarrow \alpha \;\theta_{\mathrm{ema}}+\left(1-\alpha \right)\;\theta$42:           **end if**43:     **end for**44: **end for**45: **return** θ

## 4. Experiments

### 4.1. Experimental Settings

We conduct subject-dependent motor imagery (MI) classification experiments on two public benchmarks: BCI Competition IV-2a and BCI Competition IV-2b. For IV-2a, we follow the official protocol by using the training session for model training and the testing session for evaluation. For IV-2b, we use sessions 1–3 for training and sessions 4–5 for testing. We apply no additional signal preprocessing. Each trial is constructed by directly cropping the raw EEG around the cue onset using a 4.5 s segment (0.5 s pre-cue and 4 s post-cue), resulting in T=1125 samples per trial at 250 Hz.

We evaluate the algorithms on three representative deep MI backbones: EEGNet [18], ShallowConvNet [17], and ATCNet [23]. All three models are implemented following the original architectures/hyperparameters. For ATCNet, which contains multiple parallel branches, we aggregate the branch outputs by averaging the branch logits to obtain the final logit prediction.

For each dataset–backbone pair, we construct an ensemble teacher using K-fold and bagging with K=5. Each teacher is trained for $E_{T}=750$ epochs using cross-entropy (CE) with a fixed learning rate of 0.001. All models are optimized using AdamW (weight decay 0.009) with batch size 64. Distillation hyperparameters are fixed across datasets/backbones as follows: temperature τ=4, $\lambda_{\mathrm{ens}}=0.5$, $\lambda_{\mathrm{ema}}=0.4$, $h_{\mathrm{low}}=0.6$, $h_{\mathrm{high}}=0.9$, and EMA coefficient α=0.995. Following our protocol, we select the model from the last epoch (no early stopping, no validation set is used). We do not use a separate validation set, because the number of labeled trials per subject is limited; holding out a validation split substantially reduces the effective training data and noticeably degrades the performance.

Performance is measured by classification accuracy. Each experiment is run three times, and we report the mean accuracy over the three runs. All experiments are implemented in PyTorch 2.0, Python 3.9, and executed on an NVIDIA RTX 4090 GPU.

### 4.2. Main Results

We report the subject-dependent classification accuracy on BCI Competition IV-2a and IV-2b using three backbones (EEGNet, ShallowConvNet, and ATCNet). We compare (i) the original backbone trained with standard cross entropy loss for 750 epochs, (ii) the bagging ensemble of *K* backbone models, and (iii) the proposed method that trains a single backbone student to approach the ensemble performance.

**Figure 3:**
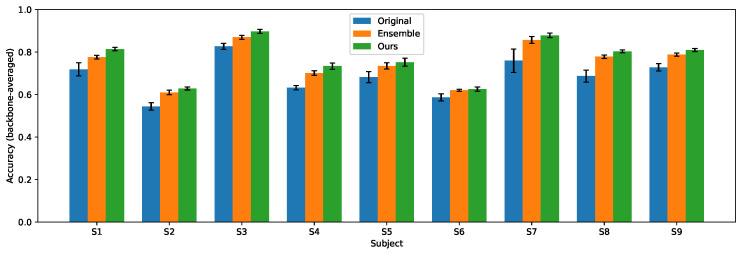
Subject-wise accuracy averaged over backbones on IV-2a.

**Figure 4:**
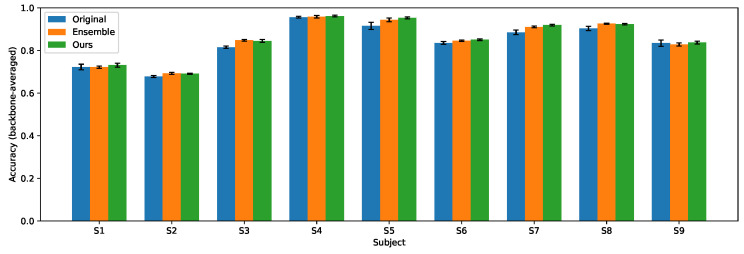
Subject-wise accuracy averaged over backbones on IV-2b.

Table 1 and Table 2 show the experiment results. Subject-wise distributions are provided in Figure 3 and Figure 4. Across both datasets and all backbones, the ensemble baseline substantially improves over the original single model, confirming that ensembling reduces the variance and enhances the robustness in MI-EEG classification. Our method substantially closes the gap between the original model and the ensemble teacher and in most cases exceeds the ensemble performance while preserving the inference cost of a single backbone. These results support the effectiveness of combining a high-quality ensemble teacher with entropy-based guidance for training compact yet accurate MI classifiers.

### 4.3. Accuracy–Latency and Accuracy–Memory Trade-Off

To evaluate the deployment-oriented efficiency, we visualize the trade-off between the classification accuracy and inference cost. For each dataset (IV-2a and IV-2b), we plot a scatter diagram, where the *x*-axis is accuracy, and the *y*-axis is the average per-trial inference latency or memory. Each point corresponds to one model variant, including the following: (i) original single backbone models (EEGNet, ShallowConvNet, ATCNet), (ii) the corresponding ensemble models formed by *K* backbones, and (iii) the proposed method (Ours) that distills ensemble knowledge into a single backbone. We measure the inference latency and GPU memory usage on a single NVIDIA RTX 4090 GPU. The batch size is set to 1 to reflect the online BCI setting where trials arrive sequentially.

**Figure 5:**
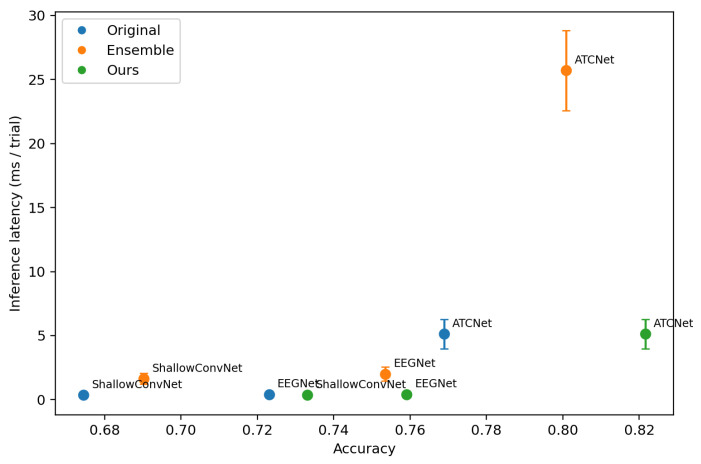
Accuracy–latency trade-off on BCI Competition IV-2a. Each point corresponds to one model configuration (Original/Ensemble/Ours) for a given backbone. Latency is measured as the mean inference time per trial on our hardware setup. Error bars indicate the standard deviation of latency measured over repeated forward passes.

**Figure 6:**
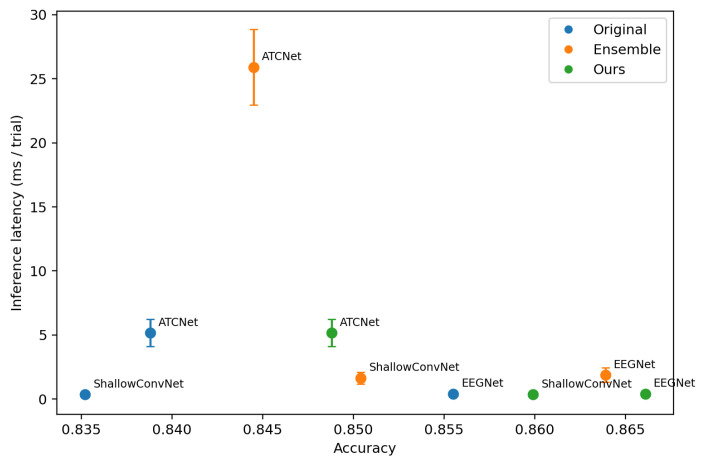
Accuracy–latency trade-off on BCI Competition IV-2b. The setting is the same as Figure 5.

**Figure 7:**
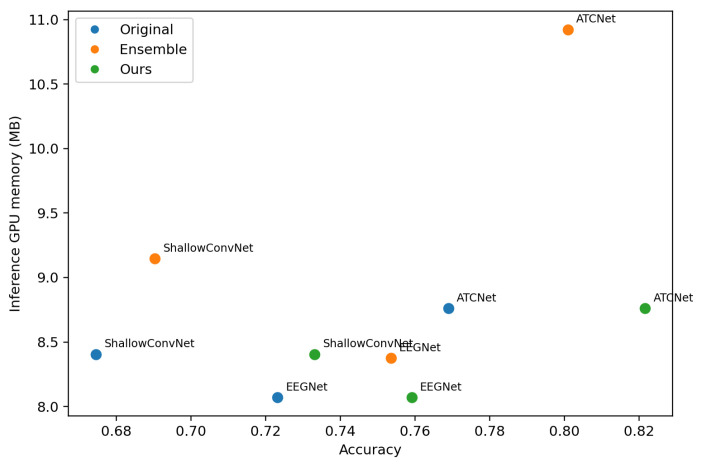
Accuracy–memory trade-off on BCI Competition IV-2a. Each point corresponds to one model configuration (Original/Ensemble/Ours) for a given backbone. Memory is measured as the mean inference memory per trial on our hardware setup.

**Figure 8:**
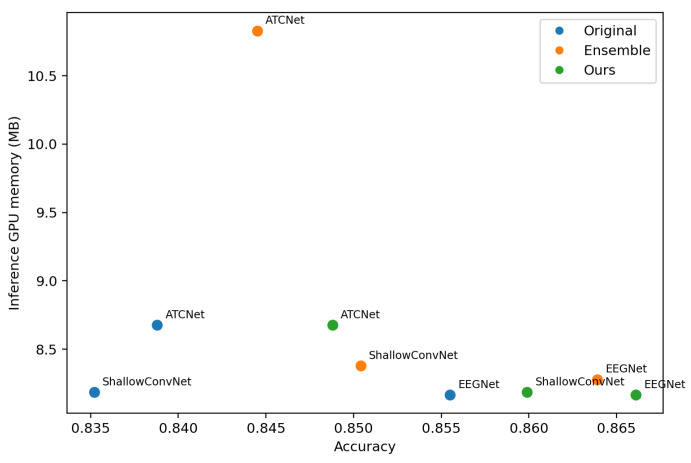
Accuracy–memory trade-off on BCI Competition IV-2b. The setting is the same as Figure 7.

Figure 5 and Figure 6 visualize the accuracy–latency trade-off across backbones and training strategies, Figure 7 and Figure 8 visualize the accuracy–memory trade-off. On IV-2a, ATCNet achieves the highest accuracy among the original single models, but it also incurs substantially higher inference latency and memory than EEGNet and ShallowConvNet. Notably, even as a single model, ATCNet already outperforms the ensemble baselines built upon EEGNet and ShallowConvNet, indicating that improving the backbone architecture is often the most effective way to boost performance when cost is not strictly constrained. However, ensembling remains unattractive for deployment due to its amplified cost. In contrast, our method consistently narrows the gap between a single student and its ensemble teacher while keeping the inference cost unchanged, making it more suitable for cost-sensitive scenarios. This effect is particularly valuable for strong backbones such as ATCNet, where further gains from architectural improvement are increasingly difficult, and using an ensemble teacher provides an effective way to inject stronger supervision.

### 4.4. Comparison to Methods in the Literature

To further evaluate the proposed method, we compare against three representative distillation paradigms widely used in deep learning: Deep Mutual Learning [26], Born-Again Networks [27], and Decoupled Knowledge Distillation [28], which cover common teacher–student distillation strategies. All methods are evaluated under the same subject-dependent protocol, datasets, backbones, and environment as in Section 4.1. The results on BCI Competition IV-2a and IV-2b are summarized in Table 3 and Table 4, respectively.

Deep Mutual Learning (DML): DML replaces the standard teacher–student transfer with collaborative learning among multiple peer models trained simultaneously. Each peer is optimized by the standard cross-entropy loss and a peer-wise KL mimicry loss that matches its predictive distribution to those of the other peers. In our implementation, we train K=5 models jointly using the peer-to-self and peer-wise KL formulation with temperature τ=4 and KL weight $\lambda_{\mathrm{dml}}=0.2$. Following the common practice for obtaining a single deployable model, we report the performance of the first peer network as the final result.

Born-Again Networks (BAN): BAN performs self-distillation across generations. A teacher is first trained using standard supervision; then, a new student with the same architecture is trained using a combination of cross-entropy and distillation from the previous generation teacher. The process can be repeated to obtain progressively improved students. We train a Generation-0 model with CE for 750 epochs and then iteratively train the next generation for 1500 epochs using CE + KD, with KD temperature τ=4 and KD weight 0.5. We repeat this procedure for four generations and report Generation-4 as the final model. We select four generations for BAN because the performance saturates after four generations.

Decoupled Knowledge Distillation (DKD): DKD reformulates the classical logit-based KD into two complementary terms, target-class KD (TCKD) and non-target-class KD (NCKD), and uses independent weights to balance the two components. In our comparison, DKD uses the same ensemble teacher as our method (an ensemble of K=5 backbones), while replacing the conventional KD loss with DKD: α=1.0 for TCKD and β=8.0 for NCKD, with temperature τ=4. α and β are selected according to the recommended configuration from the original DKD paper.

Table 3 and Table 4 show that our method achieves the best average performance on both datasets, consistently outperforming DML, BAN, and DKD across the three backbones. Notably, the relative advantages of the literature methods vary with the backbone strength. In contrast, our method yields the most robust improvements.

### 4.5. Ablation Analysis

We conduct ablation studies to isolate the contribution of each component in the proposed training pipeline. All ablations follow the same subject-dependent protocol, datasets, backbones (EEGNet, ShallowConvNet, ATCNet), and environment as the full method. The following ablations are compared:

**(A1) Ours w/o Ensemble KD (EMA-only KD).** We remove the ensemble distillation term and keep only the EMA teacher guidance. The loss is reduced to $L=L_{ce}+\lambda_{\mathrm{ema}}w\left(x\right)L_{kd}^{\mathrm{ema}}$, where the EMA teacher is initialized and activated at epoch 501 (Phase II) as in the full method.

**(A2) Ours w/o EMA KD (Ensemble-only KD).** We disable the entropy-gated EMA distillation branch and retain only ensemble distillation. The loss becomes $L=L_{ce}+\lambda_{\mathrm{ens}}L_{kd}^{\mathrm{ens}}$ throughout training.

**(A3) Ours w/o Cosine Annealing.** We replace the two-stage cosine schedule with a fixed learning rate of 0.001 for the student. The EMA teacher is still activated at epoch 501, ensuring that only the learning-rate schedule is changed.

**(A4) Ours w/o entropy-gate.** We remove the entropy-gate while preserving the EMA KD. The loss becomes $L=L_{\mathrm{ce}}+\lambda_{\mathrm{ens}}L_{\mathrm{kd}}^{\mathrm{ens}}+\lambda_{\mathrm{ema}}L_{\mathrm{kd}}^{\mathrm{ema}}$ throughout training.

The results are summarized in Table 5 and Table 6. Overall, each component contributes positively, with the full method consistently achieving the best performance across datasets and backbones.

### 4.6. Predictive Entropy Analysis

This section provides an entropy-based validation of our design. Motivated by an information theoretic view of training instability, our method is explicitly designed to (i) stabilize the teacher signal via entropy-gated EMA filtering and thereby reduce the student’s predictive noise and (ii) suppress oscillations near convergence via the two-stage cosine schedule. To verify that these objectives are indeed achieved in practice, we analyze the epoch-wise dynamics of predictive entropy on a representative configuration: ATCNet on BCI Competition IV-2a, Subject 2, using a single run. We compare our method (EMA+Cosine) with two ablations that isolate each component: A2 (NoEMA+Cosine) to evaluate the effect of EMA filtering on entropy level and A3 (EMA+NoCosine) to evaluate the effect of cosine annealing on entropy fluctuation.

Given the predicted class probability vector $p\left(x\right)\in R^{C}$ for a test sample x, we compute the predictive entropy as

(15) $$H\left(x\right)=-\sum_{c=1}^{C}p_{c}\left(x\right)\log p_{c}\left(x\right),$$

where larger entropy indicates higher uncertainty, and smaller entropy indicates a more peaked predictive distribution. We conduct the following three experiments:

(1) Entropy trajectories for correct vs. incorrect predictions.

For each epoch *e*, we evaluate the student model on the test set and partition test samples into correctly classified and misclassified subsets, denoted by $T_{e}^{\mathrm{corr}}$ and $T_{e}^{\mathrm{err}}$. We then compute the mean predictive entropy for each subset:

(16) $${\bar{H}}^{\mathrm{corr}}\left(e\right)=\frac{1}{|T_{e}^{\mathrm{corr}}|}\sum_{x\in T_{e}^{\mathrm{corr}}}H\left(x\right),\;{\bar{H}}^{\mathrm{err}}\left(e\right)=\frac{1}{|T_{e}^{\mathrm{err}}|}\sum_{x\in T_{e}^{\mathrm{err}}}H\left(x\right).$$

**Figure 9:**
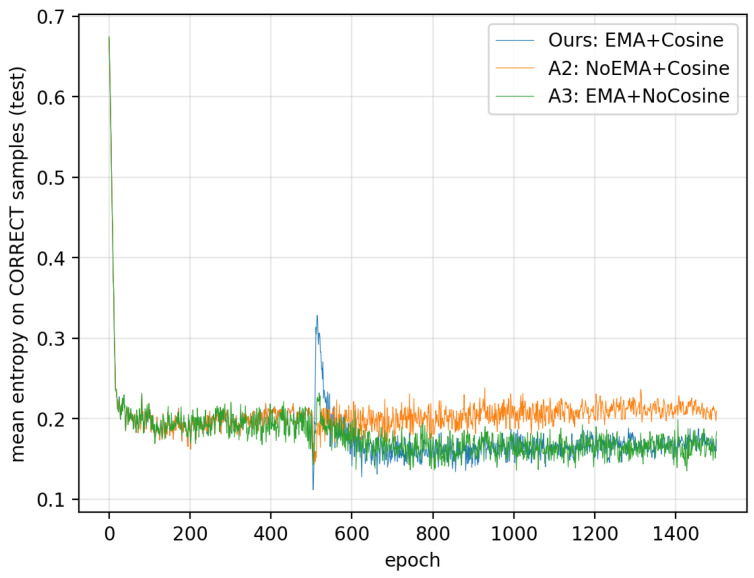
Mean entropy on correct samples.

**Figure 10:**
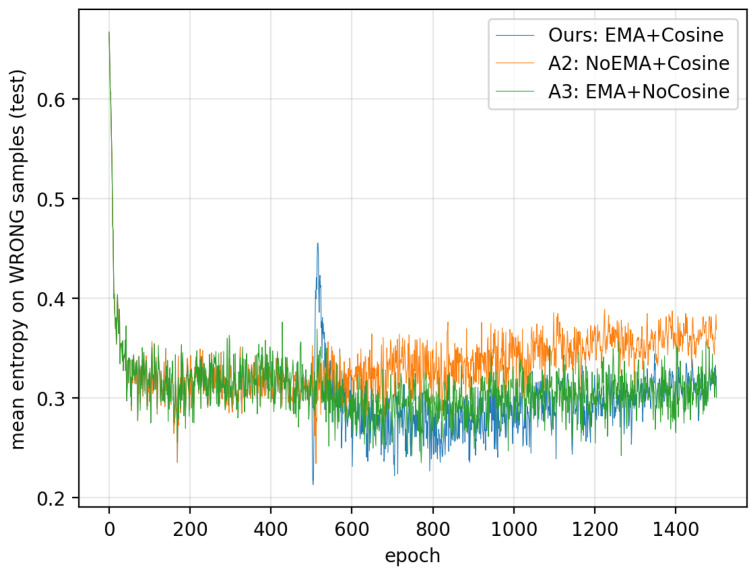
Mean entropy on wrong samples.

We visualize ${\bar{H}}^{\mathrm{corr}}\left(e\right)$ and ${\bar{H}}^{\mathrm{err}}\left(e\right)$ as two curves across epochs (Figure 9 and Figure 10). A clear trend emerges after Phase II begins (EMA enabled): compared with A2, both the correct-sample entropy and the wrong-sample entropy are consistently lower under the EMA-enabled settings (A3 and Ours).

The EMA teacher can be viewed as a low-pass filter in parameter space, which yields temporally smoothed teacher logits and more stable soft targets. Logit-level instability can be modeled as non-common-mode perturbations that change relative logit differences (e.g., per-class additive noise), which tends to flatten the predictive distribution and increase the predictive entropy on average. By distilling from the EMA teacher, the student receives a less noisy guidance signal, which stabilizes the student logits. Consequently, the overall predictive entropy on the test set decreases after EMA activation, as observed in Figure 9 and Figure 10. The entropy decrease across datasets and backbones is shown in Table 7.

(2) Entropy fluctuation over a sliding window.

In the second experiment, we quantify how much the model’s prediction fluctuates across epochs. For each test sample x, we compute a sliding-window variance of its predictive entropy over the current epoch and the previous 19 epochs (window size W=20):

(17) $${\mathrm{Var}}_{W}\left(x,e\right)=\mathrm{Var}\left({\left\{H\left(x,i\right)\right\}}_{i=\max(1,e-W+1)}^{e}\right).$$

**Figure 11:**
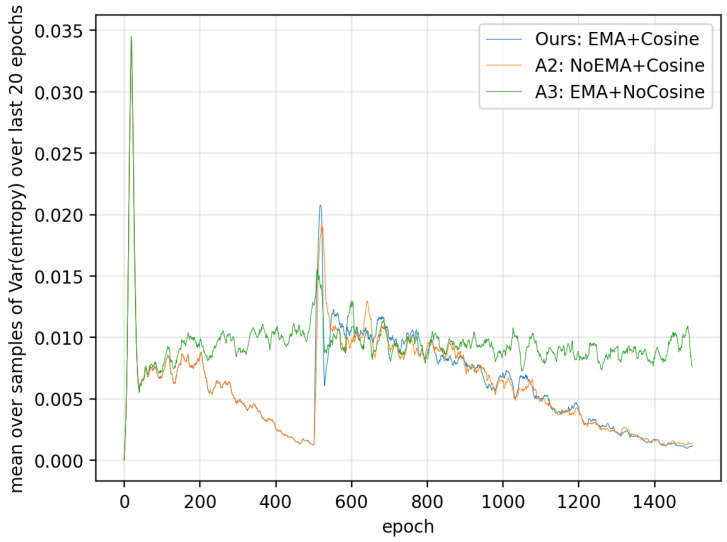
Mean over samples of entropy variance over last 20 epochs.

We then average this quantity over all test samples to obtain an epoch-wise entropy fluctuation score:

(18) $${\bar{\mathrm{Var}}}_{W}\left(e\right)=\frac{1}{\left|T\right|}\sum_{x\in T}{\mathrm{Var}}_{W}\left(x,e\right),$$

where T denotes the test set. We plot ${\bar{\mathrm{Var}}}_{W}\left(e\right)$ across epochs to visualize the stability of the predictive entropy during training. Figure 11 reports the results. In the late stage of training, A3 exhibits noticeably larger fluctuation than both Ours and A2, which enable the cosine schedule. This indicates that the cosine annealing schedule plays a dominant role in suppressing late-stage oscillations. Practically, reduced fluctuation makes the final checkpoint selection more reliable, because the model behavior near convergence becomes more stable across epochs. The entropy variance decrease across datasets and backbones is shown in Table 8.

(3) Correlation analysis between entropy decrease and accuracy increase.

To quantify the relationship between entropy decrease and performance change across subjects, we perform an correlation analysis for each dataset–backbone setting.

For each dataset and backbone, we treat each subject *s* as one sample point and get the accuracy $Acc_{s}^{m}$, mean entropy across test samples $H_{s}^{m}$, and mean entropy variance across test samples $Var_{s}^{m}$ under method m∈{Ours,A2,A3} at the end of training. We define the subject-wise changes relative to a baseline method *b* as

(19) $$\Delta Acc_{s}^{b}=Acc_{s}^{\mathrm{Ours}}-Acc_{s}^{b},\;\Delta H_{s}^{b}=H_{s}^{b}-H_{s}^{\mathrm{Ours}},\;\Delta {Var}_{s}^{b}={Var}_{s}^{b}-{Var}_{s}^{\mathrm{Ours}},$$

We then compute the Pearson correlation between $\Delta Acc_{s}^{b}$ and $\Delta H_{s}^{b}$ (also $\Delta {Var}_{s}^{b}$).

We report the resulting correlations in Table 9. The results show a positive correlation in general.

## 5. Discussion

This work targets a practical tension in MI-EEG decoding: achieving ensemble-level performance while keeping single-model inference cost for real-time or edge BCI deployment. Across two public benchmarks and three representative backbones, the proposed entropy-based dual-teacher distillation consistently improves a deployable single model student and, in most cases, approaches or exceeds the corresponding ensemble teacher.

We interpret MI-EEG training instability from an information-theoretic perspective, with predictive entropy serving as a measurable proxy of uncertainty in the predictive distribution. Under this view, the offline ensemble teacher primarily improves the quality of soft supervision, while the EMA teacher plays a complementary role as a denoising mechanism. Due to the low signal-to-noise ratio and limited per-subject data, MI training is often sensitive to random initialization and optimization noise, and the resulting logits may exhibit non-common-mode perturbations that change relative logit differences across classes. Such perturbations tend to flatten the predictive distribution, leading to higher predictive entropy. Distilling from an EMA teacher therefore transfers a more stable supervisory signal to the student, which is reflected as a lower mean predictive entropy on the test set. To further connect this stability proxy to performance, we additionally report correlation studies between entropy-based changes and accuracy changes, showing that entropy reduction captures improved stability even when accuracy gains vary across subjects due to strong inter-subject variability. It is important to note that the role of entropy in this work is different from probability calibration: we use predictive entropy primarily as a proxy for logit-level noise under scarce and noisy MI data, and our entropy gate relies on predictive entropy to distinguish high-noise vs. low-noise samples, rather than requiring well-calibrated probabilities.

The entropy-gated activation further improves robustness by allocating EMA guidance adaptively across samples. We increase the weights of samples with higher entropy and decrease the weights of low-entropy samples (easy/confident cases) to avoid redundant regularization. This weighting concentrates the stabilization effect where it is most needed, while preserving discriminative learning on easy samples. Complementarily, the two-stage cosine annealing schedule suppresses late-stage oscillations, which manifests as reduced entropy fluctuation near convergence, making checkpoint selection more reliable.

The results suggest a simple “train heavy, infer light” recipe for MI-EEG: use a strong but offline ensemble teacher to provide high-quality supervision, and stabilize the student with entropy-based mechanisms to obtain a deployable single model with ensemble-like performance. This is particularly useful for strong backbones (e.g., ATCNet), where further architectural modifications may yield diminishing returns; in such cases, an ensemble teacher becomes a practical way to strengthen supervision. More broadly, the proposed framework is backbone-agnostic and can be integrated into existing MI-EEG pipelines with minimal changes.

## 6. Conclusions

This paper presented a dual-teacher distillation framework for efficient motor-imagery EEG classification under practical latency constraints. The proposed approach distills knowledge from an offline ensemble teacher and introduces an entropy-gated EMA teacher that acts as a low-pass filter on parameters to produce denoised guidance. Once activated in the second stage of cosine annealing, the EMA-guided distillation transfers stability to the student, resulting in a denoised predictive distribution. Moreover, the entropy-gated weighting modulates the EMA KD term at the sample level, emphasizing uncertain samples and deemphasizing easy samples to focus the denoising effect where it is most beneficial. Complementarily, the two-stage cosine annealing schedule reduces late-stage fluctuation, making convergence behavior and checkpoint selection more reliable. Experiments on BCI Competition IV-2a and IV-2b with three representative backbones demonstrated that the proposed method consistently closes the performance gap to ensembles, while avoiding the inference-time overhead incurred by multi-member ensembles.

Future work will extend this framework to subject-independent settings and explore richer distillation (e.g., network intermediate representations) to further improve the robustness in real-world BCI deployments.

## Figures and Tables

**Table 1:** Subject-dependent accuracy on BCI Competition IV-2a. Results are averaged over nine subjects. We report mean accuracy and *p*-values (in the bracket) from subject-level Wilcoxon signed-rank tests comparing our method vs. baseline on per-subject scores (two-sided). * indicates *p* < 0.05, ** indicates *p* < 0.01. Bolds indicate the highest accuracy for each setting.

Backbone	Original	Ensemble (*K* Models)	Ours (Single Student)
EEGNet	0.7231 (** 0.0039)	0.7535 (0.1230)	**0.7591**
ShallowConvNet	0.6745 (** 0.0039)	0.6903 (** 0.0039)	**0.7331**
ATCNet	0.7689 (** 0.0039)	0.8009 (* 0.0195)	**0.8216**
**Average**	0.7222 (** 0.0039)	0.7482 (** 0.0039)	**0.7713**

**Table 2:** Subject-dependent accuracy on BCI Competition IV-2b. Results are averaged over nine subjects. We report mean accuracy and *p*-values (in the bracket) from subject-level Wilcoxon signed-rank tests comparing our method vs. baseline on per-subject scores (two-sided). * indicates *p* < 0.05, ** indicates *p* < 0.01. Bolds indicate the highest accuracy for each setting.

Backbone	Original	Ensemble (*K* Models)	Ours (Single Student)
EEGNet	0.8555 (** 0.0039)	0.8639 (0.5735)	**0.8661**
ShallowConvNet	0.8352 (** 0.0039)	0.8504 (* 0.0499)	**0.8599**
ATCNet	0.8388 (0.3594)	0.8445 (0.2500)	**0.8488**
**Average**	0.8432 (** 0.0039)	0.8529 (0.0742)	**0.8583**

**Table 3:** Subject-dependent accuracy on BCI Competition IV-2a. We report mean accuracy and *p*-values (in the bracket) from subject-level Wilcoxon signed-rank tests comparing our method vs. baseline on per-subject scores (two-sided). * indicates *p* < 0.05, ** indicates *p* < 0.01. Bolds indicate the highest accuracy.

Backbone	DML	BAN	DKD	Ours
EEGNet	0.7420 (** 0.0078)	0.7441 (* 0.0117)	0.7477 (* 0.0273)	**0.7591**
ShallowConvNet	**0.7428** (0.4257)	0.7224 (* 0.0195)	0.6993 (* 0.0273)	0.7331
ATCNet	0.7903 (** 0.0039)	0.8013 (* 0.0117)	0.8086 (** 0.0039)	**0.8216**
**Average**	0.7584 (0.1289)	0.7559 (* 0.0195)	0.7519 (* 0.0039)	**0.7713**

**Table 4:** Subject-dependent accuracy on BCI Competition IV-2b. We report mean accuracy and *p*-values (in the bracket) from subject-level Wilcoxon signed-rank tests comparing our method vs. baseline on per-subject scores (two-sided). * indicates *p* < 0.05, ** indicates *p* < 0.01. Bolds indicate the highest accuracy.

Backbone	DML	BAN	DKD	Ours
EEGNet	0.8621 (0.2070)	0.8625 (0.7794)	0.8639 (0.5754)	**0.8661**
ShallowConvNet	0.8464 (0.0742)	0.8559 (0.8203)	0.8501 (* 0.0179)	**0.8599**
ATCNet	0.8406 (* 0.0356)	0.8393 (* 0.0390)	0.8433 (0.1234)	**0.8488**
**Average**	0.8497 (** 0.0039)	0.8526 (0.4257)	0.8524 (* 0.0195)	**0.8583**

**Table 5:** Ablation results (accuracy) on BCI Competition IV-2a under the subject-dependent protocol. We report mean accuracy and *p*-values (in the bracket) from subject-level Wilcoxon signed-rank tests comparing our method vs. baseline on per-subject scores (two-sided). * indicates *p* < 0.05, ** indicates *p* < 0.01. Bolds indicate the highest accuracy.

Backbone	EMA-Only KD	Ensemble-Only KD	w/o Cosine	w/o Entropy-Gate	Ours (Full)
EEGNet	0.7422 (* 0.0195)	0.7515 (0.3593)	0.7545 (0.3593)	0.7582 (0.7794)	**0.7591**
ShallowConvNet	0.7021 (** 0.0039)	0.7255 (0.3007)	0.7253 (0.1289)	0.7112 (** 0.0039)	**0.7331**
ATCNet	0.7742 (** 0.0039)	0.7993 (* 0.0117)	0.7941 (** 0.0039)	0.7985 (** 0.0039)	**0.8216**
**Average**	0.7395 (** 0.0039)	0.7588 (** 0.0078)	0.7580 (* 0.0195)	0.7560 (* 0.0117)	**0.7713**

**Table 6:** Ablation results (accuracy) on BCI Competition IV-2b under the subject-dependent protocol. We report mean accuracy and *p*-values (in the bracket) from subject-level Wilcoxon signed-rank tests comparing our method vs. baseline on per-subject scores (two-sided). * indicates *p* < 0.05, ** indicates *p* < 0.01. Bolds indicate the highest accuracy.

Backbone	EMA-Only KD	Ensemble-Only KD	w/o Cosine	w/o Entropy-Gate	Ours (Full)
EEGNet	0.8525 (* 0.0195)	0.8598 (0.4257)	0.8622 (0.5754)	0.8645 (0.6523)	**0.8661**
ShallowConvNet	0.8407 (** 0.0039)	0.8590 (0.1797)	**0.8612** (0.7343)	0.8530 (0.8203)	0.8599
ATCNet	0.8421 (0.1230)	0.8334 (* 0.0195)	0.8357 (** 0.0078)	0.8377 (* 0.0195)	**0.8488**
**Average**	0.8451 (** 0.0078)	0.8507 (0.0546)	0.8530 (0.3593)	0.8517 (0.6523)	**0.8583**

**Table 7:** Predictive entropy decrease (averaged over all subjects) at the end of training.

Dataset	Backbone	Ours vs. A2	A3 vs. A2
	EEGNet	0.0373	0.0234
2a	ShallowConvNet	0.0431	0.0457
	ATCNet	0.0151	0.0196
	EEGNet	0.0262	0.0351
2b	ShallowConvNet	0.0366	0.0372
	ATCNet	0.0426	0.0549

**Table 8:** Entropy variance decrease (averaged over all subjects) at the end of training.

Dataset	Backbone	Ours vs. A3	A2 vs. A3
	EEGNet	0.0021	0.0025
2a	ShallowConvNet	0.0055	0.0052
	ATCNet	0.0082	0.0086
	EEGNet	0.0017	0.0014
2b	ShallowConvNet	0.0051	0.0059
	ATCNet	0.0063	0.0077

**Table 9:** Correlation between entropy (also entropy variance) decrease and accuracy increase.

Dataset	Backbone	Entropy	Entropy Variance
	EEGNet	0.1980	−0.2456
2a	ShallowConvNet	0.0686	0.3986
	ATCNet	0.5496	0.7095
	EEGNet	0.0059	−0.0268
2b	ShallowConvNet	0.0499	−0.2893
	ATCNet	0.4549	0.5125

## Data Availability

The BCI Competition IV 2a and 2b datasets utilized in this study are publicly accessible. To access it, visit following website: http://bnci-horizon-2020.eu/database/data-sets (accessed on 12 January 2026).
